# Expanding the Cell-Free Reporter Protein Toolbox by Employing a Split mNeonGreen System to Reduce Protein Synthesis Workload

**DOI:** 10.1021/acssynbio.3c00752

**Published:** 2024-06-05

**Authors:** Caroline E. Copeland, Chloe J. Heitmeier, Khoa D. Doan, Shea C. Lee, Kassidy B. Porche, Yong-Chan Kwon

**Affiliations:** † Department of Biological and Agricultural Engineering, Louisiana State University, Baton Rouge, Louisiana 70803, United States; ‡ Louisiana State University Agricultural Center, Baton Rouge, Louisiana 70803, United States

**Keywords:** Cell-free biosensor, Split green fluorescent protein, SynZip, mNeonGreen

## Abstract

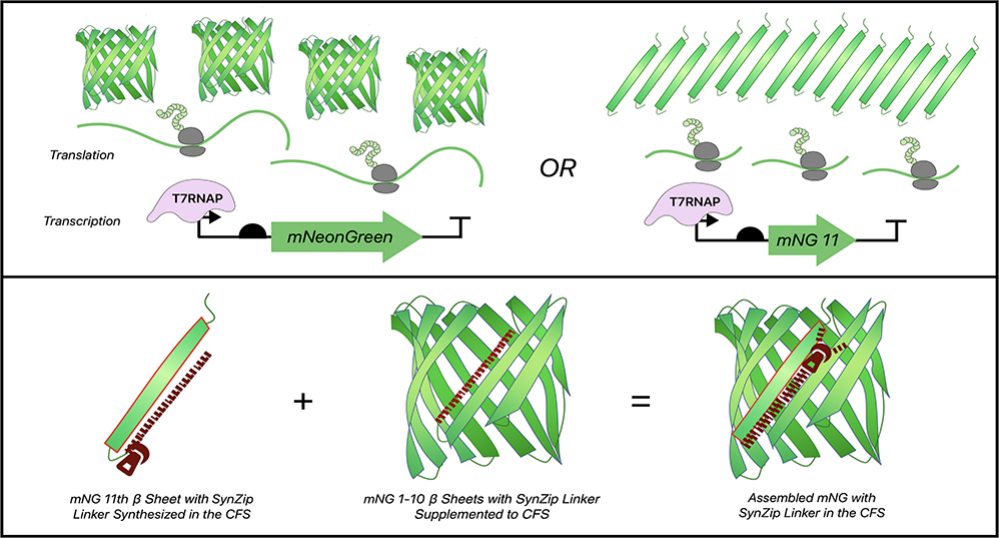

The cell-free system offers potential advantages in biosensor applications, but its limited time for protein synthesis poses a challenge in creating enough fluorescent signals to detect low limits of the analyte while providing a robust sensing module at the beginning. In this study, we harnessed split versions of fluorescent proteins, particularly split superfolder green fluorescent protein and mNeonGreen, to increase the number of reporter units made before the reaction ceased and enhance the detection limit in the cell-free system. A comparative analysis of the expression of 1–10 and 11th segments of beta strands in both whole-cell and cell-free platforms revealed distinct fluorescence patterns. Moreover, the integration of SynZip peptide linkers substantially improved complementation. The split protein reporter system could enable higher reporter output when sensing low analyte levels in the cell-free system, broadening the toolbox of the cell-free biosensor repertoire.

## Introduction

The cell-free system (CFS) has proved to be an ideal system for biosensor design and development, leveraging its open architecture, selectivity for a specific pathway of interest, and cellular pathway mimicry to facilitate precise sensing mechanisms.^1^ While cell-free biosensors harness complex gene circuits for refined biosensing, the CFS is limited in its protein expression time period.^2^ Therefore, larger reporter proteins that require more time to be made will result in fewer fluorescent units and lower fluorescence for low concentrations of analytes. In this context, we integrated the split fluorescent protein system into the CFS, aiming for a smaller-size biosensor reporter to result in more units synthesized in the system and reach lower limits of detection by allowing more reporter protein to be made.

Split fluorescent proteins, such as superfolder green fluorescent protein (sfGFP)^3−5^ and mNeonGreen (mNG),^6,7^ fused with the proteins of interest, have previously been used to visualize protein–protein interactions. Typically, the split fluorescent protein application involves splitting the 11th β-strand from the 11-segment β-stranded barrel of the green fluorescent protein and fusing them onto the proteins of interest. In this study, the small 11th β-strand is expressed independently, without being fused to a larger protein as is commonly done, resulting in intriguing findings. The reconstitution of fluorescent signal is spontaneous, without a covalent linkage, facilitated by its barrel-shaped protein’s tolerance to circular permutation.^8^ This study sought to express the small 11th β-strand (17 amino acids long, 2.0 (sfGFP) – 2.2 (mNG) kDa) as the reporter of the sensor without being fused to a larger protein, which allows the system to synthesize more units of the reporter compared to synthesizing full-length sfGFP (230 amino acids long, 25.8 kDa) and mNG (227 amino acids long, 25.7 kDa) as the reporter. The overexpressed and purified large segment of the fluorescent protein (first-tenth β-strands (1–10)) would be supplemented into the cell-free reaction prior to activation of the sensor. The full-length sfGFP and mNG have previously been characterized in the CFS as bright reporter protein candidates.^9^ For the split mNG used in this study, we particularly utilized the complementation-enhanced version of the split mNG to maintain complementation capability even at low expression levels.^7^

In this study, we elucidated the feasibility of the split protein interaction compared to the whole-cell system and explored the complementation efficiencies and brightness between sfGFP and mNG split protein systems in the CFS. In addition, the gene ratios of the 1–10 and 11th segments were investigated to enhance the output signal. Moreover, we improved the complementation of mNG split segments by adding SynZip peptide linkers^10^ and demonstrated the expansion of the cell-free biosensor toolbox with lower limits of detection.

## Results and Discussion

### Difference in Complementation Efficiencies across Fluorescent Protein and Expression Systems

**Figure 1:**
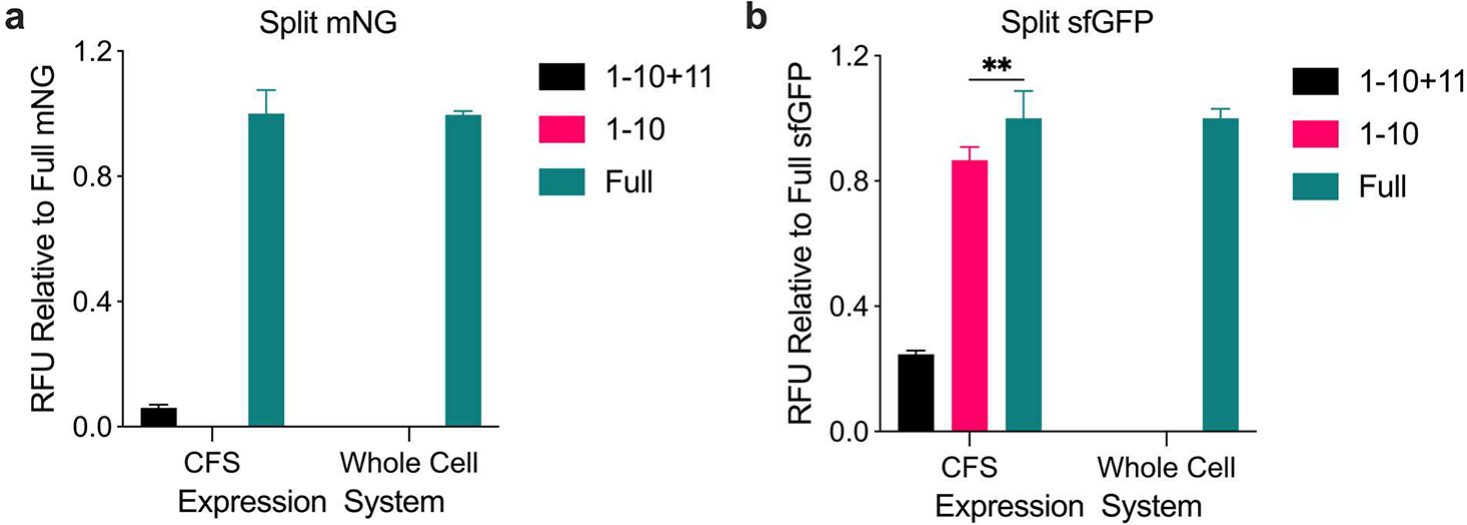
Split fluorescent protein expression in two protein expression systems. a) Split mNG expression in the CFS and whole-cell culture compared to full-length mNG. b) Split sfGFP expression in the CFS and whole-cell culture compared to full-length sfGFP. Black bar: coexpression of the 1–10 and the 11th segments, Red bar: expression of the 1–10 segment alone, Green bar: full-length protein expression. Values represented as mean ± SD, *n* = 3, **p < 0.01.

The 11th segments for both sfGFP and mNG were cloned into the pJL1 vector for cell-free reaction. For preliminary expression and fluorescence screening purposes, the 1–10 segments were also cloned into the same vector and coexpressed with the 11th segment in the CFS to avoid potential low protein synthesis in the CFS using pET vectors.^11^ For whole-cell expression, the 1–10 segment genes were cloned in the pETBlue1 vector, ensuring their overexpression by growing them in the presence of antibiotics. Both full-length (unsplit) sfGFP and mNG were synthesized completely, generating the fluorescent in both cell-free and whole-cell expression systems (Figure 1). When 1–10 segments were expressed in the CFS, the sfGFP 1–10 segment exhibited high fluorescence compared to its cell-free synthesized full-length counterpart, while the mNG 1–10 segment did not develop the fluorescence (Figure 1). This phenomenon complicated the analysis of split sfGFP complementation in the CFS, especially since introducing the 11th segment resulted in decreased fluorescence than both the 1–10 segments and full-length proteins (Figure 1b, black bar). The phenomenon of sfGFP 1–10 fluorescing on its own has never been seen before because no one has expressed this construct in the CFS. Sequencing was performed on the plasmid to confirm that it was truly the truncated sfGFP that was being expressed. In contrast, the mNG 1–10 segment alone did not develop fluorescence on its own in the CFS. However, mNG did develop a small fluorescence increase in the presence of the 11th segment (coexpression of 1–10 and 11th segments) (Figure 1a, black bar). The expected higher fluorescent output observed with the split mNG 1–10 was not observed in this case, likely because the 11th segment was expressed independently, without being fused to a larger complex that would help stabilize the 11th segment and prevent its degradation. This might have reduced the complementation efficiency. To investigate whether the inherent fluorescence of the sfGFP 1–10 segment resulted from its expression in the CFS, we transitioned the split expression to a whole cell expression system. In the whole-cell system, neither the 1–10 segment alone nor its coexpression with the 11th segment exhibited fluorescence, indicating the split mNG system is better aligned with the CFS (Figure 1). The difference in fluorescence between the 1–10 segments of sfGFP and mNG could be attributed to sfGFP being a weak dimer, whereas mNG exists as a monomer.

### Varying Gene Concentrations of the Split Protein Segments

**Figure 2:**
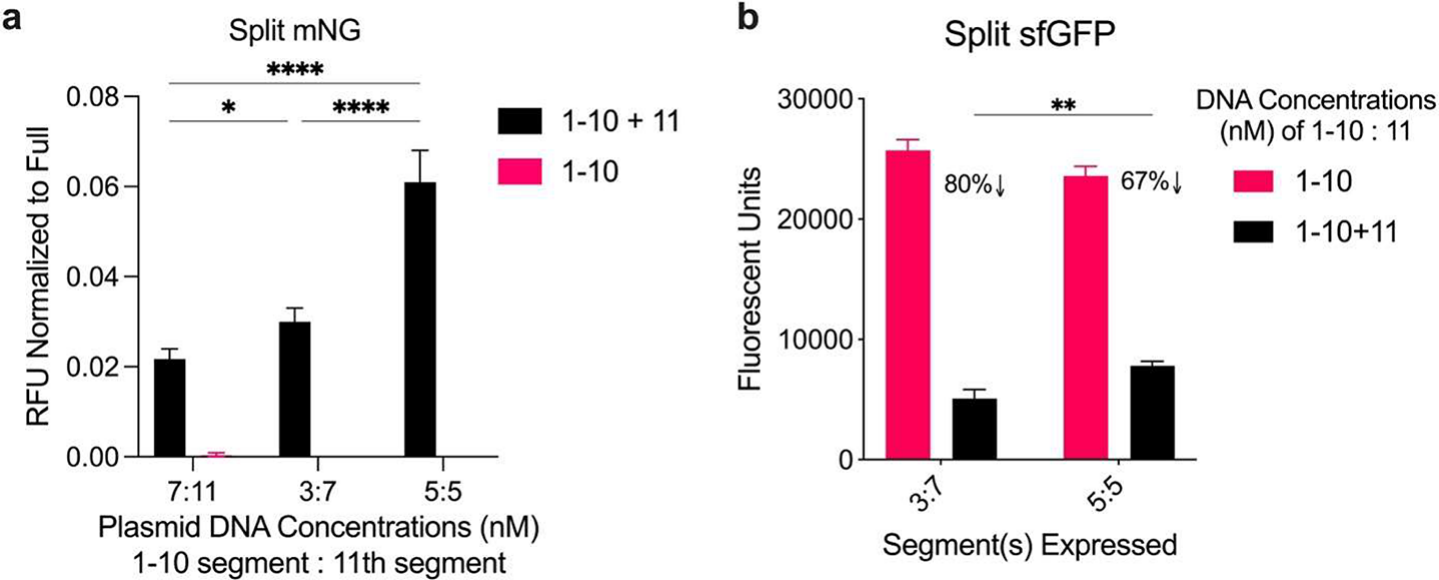
Varying DNA concentration ratio of the two split segments in the CFS. a) Split mNG expression values normalized to full-length mNG supplied at the same concentration as the 1–10 segment plasmid DNA. b) Split sfGFP expression absolute values at varying DNA concentrations. Black bar: 1–10 segment DNA ratio, Red bar: 11th segment DNA ratio. Values represented as mean ± SD, *n* = 3, *p < 0.05, **p < 0.01, ****p < 0.0001.

Next, we investigated the gene concentrations of the split protein segments. The DNA concentrations in the CFS were varied to determine two objectives: 1) to ascertain the optimal ratio between the 1–10 segment gene and the 11th segment gene for the effective functioning of the split mNG system and 2) to differentiate whether the 11th segment of the sfGFP was interfering with the folding of 1–10 segment of sfGFP or facilitating its assembly. We found that an equimolar ratio between 1 and 10 and the 11th segment gene was optimal for the split mNG system. However, the equimolar ratio did not enhance the brightness up to the level of the full-length protein. The data is normalized to the fluorescent output from full mNG expressed from plasmid DNA supplemented at the same concentration as the 1–10 segment DNA concentration listed (Figure 2a). For the split sfGFP system, an equimolar amount of the split segment genes (5:5) showed a lower drop (67%) in fluorescence of the 1–10 segment (fluorescing on its own, as discovered earlier) than the 3 nM 1–10 to 7 nM of 11 (80%) (Figure 2b). Comparing these two data points where the total plasmid concentration is equal implies that the 11th segment might be impeding the expression of the 1–10 segment rather than aiding in its assembly.

#### Improving Split mNG Interaction with SynZip Peptide Linkers

To enhance the conjoining of the 11th and large 1–10 segments, we incorporated linker peptides known as SynZip 17 and 18 on each respective segment. These SynZip linkers have previously demonstrated their efficacy in improving split T7 RNA polymerase assemblies,^12^ and we observed similar enhancement in this study. The SynZip 17 sequence (Table S1) was added to the C-terminus of the mNG 1–10 segment gene in the pETBlue1 vector. The large mNG 1–10 segment was then expressed in *Escherichia coli* strain BL21(DE3) Star to facilitate protein purification using the N-terminal histidine tag (Figure S1). The purified large mNG segment was supplied to the CFS. In contrast, the SynZip 18 sequence (Table S1) was integrated at the N-terminus of the 11th segment in the pJL1 vector and expressed in the CFS, increasing the peptide size from 2.2 to 7.3 kDa.

**Figure 3:**
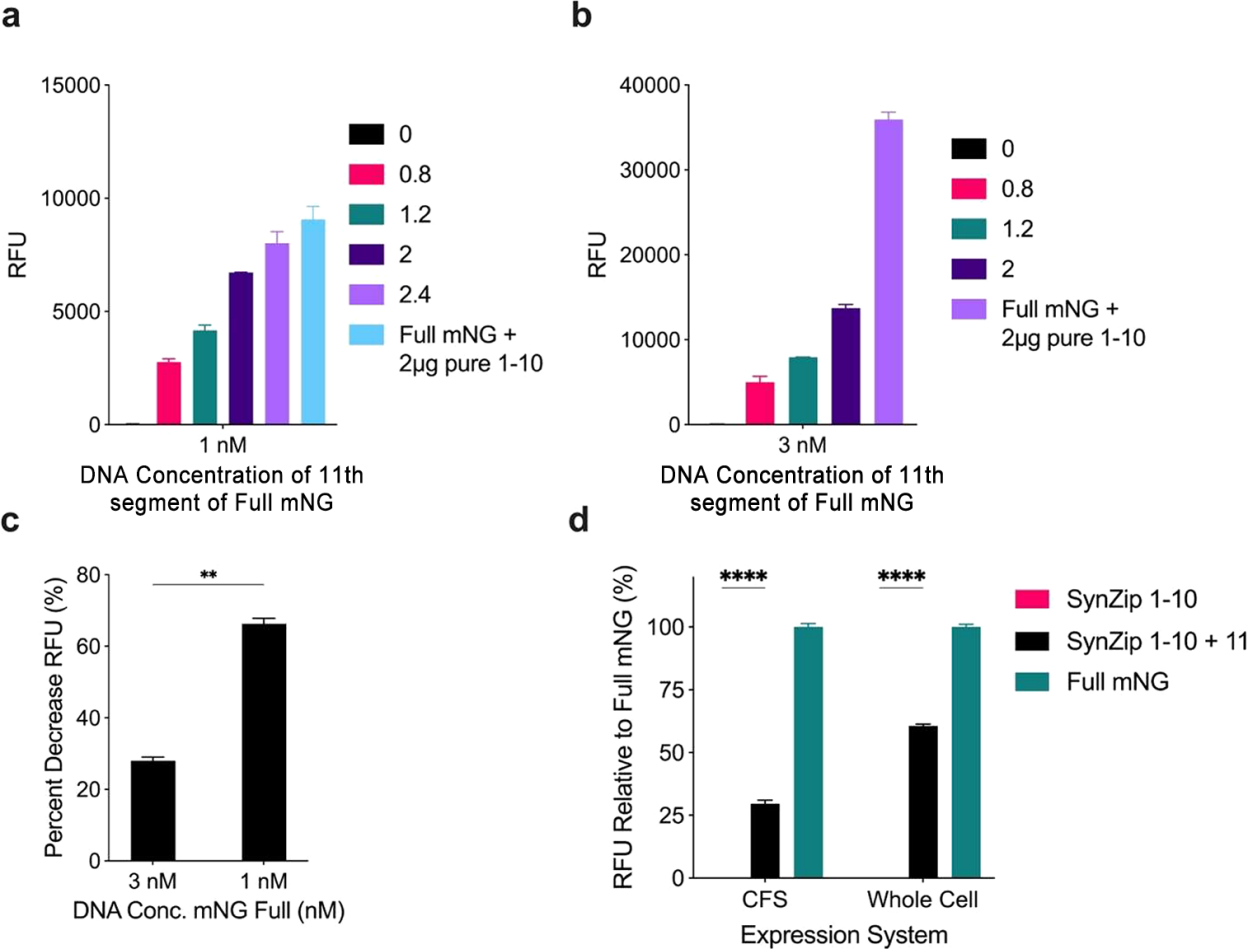
Improvement of split mNG with the addition of SynZip linkers. a) Fluorescent output of the CFS with 1 nM DNA concentration and varying purified mNG 1–10 segment concentrations. b) Fluorescent output of the CFS with 3 nM DNA concentration and varying purified mNG 1–10 segment concentrations. c) Percent decrease in protein expression when 2 μg of purified mNG 1–10 segment is added to the CFS expressing full-length mNG at varying concentrations. d) Split mNG expression in the CFS and whole-cell culture compared to full-length mNG with SynZip linkers. Values represented as mean ± SD, *n* = 3, **p < 0.01, ****p < 0.0001.

To better represent the split system as a reporter, the DNA concentration of the 11th segment was kept at an equal amount as the full since a cell-free sensor would trigger both reporters at the same strength, with only the natural decline in protein synthesis dictating the amount of protein made at that point. To test the split mNG with the SynZip linker, the amount of purified 1–10 segment was varied, along with the DNA concentration of the 11th segment in the CFS (Figure 3a and 3b). The success of complementation was notably higher when observed at the lower concentration of 1 nM of the 11th segment DNA, achieving a fluorescence signal representing 73.6% ± 0.2% of the full-length mNG under the same conditions. However, it was noticed that the addition of the purified 1–10 segment did inhibit protein expression of the reaction overall, leading to a decrease of 28.0% ± 1% at 3 nM DNA concentration of full-length mNG and a 66.3% ± 1.5% decrease at 1 nM (Figure 3c). Potential causes for this reduction in expression could be the trace proteins in the purified product or the possibility that the isolated 1–10 segment may aggregate in the absence of its 11th segment counterpart, thereby interfering with the stoichiometric balance of the other proteins in the CFS.^3,5^

For instance, in Figure 3c, a notable decrease in protein production was observed at a lower DNA concentration of 1 nM compared to 3 nM. At 1 nM, the mRNA in the system is not in excess, so any hindrance to the transcriptional machinery can be more dramatically seen through the protein output. If there was an excess in mRNA, then a hindrance to transcription would not affect the protein output as much due to the system having enough mRNA until it reaches the end point. This can be seen when you increase the DNA concentration, allowing the system to have more mRNA to work with, and showing a lower percent decrease in expression of the full mNG fluorescence. If the purified protein and trace proteins are affecting the transcription module, it could be affecting more aspects of the system, as well as the complementation efficiency.

Nevertheless, the addition of SynZip linkers enhanced the fluorescence of the conjoined split mNG, elevating it by 4.8 ± 0.2-fold in the CFS and allowing for fluorescence to occur in general in the whole-cell expression system, surpassing the performance in the CFS (Figure 3d).

## Conclusion

Here, we have debuted the split fluorescent protein system in the CFS for the first time. The sfGFP 1–10 segment alone exhibited fluorescence in the CFS but remained inactive in a whole-cell system. This could potentially suggest that the cell-free environment supports the improved protein folding dynamics of the incomplete structure of the sfGFP 1–10 segment. However, this feature also proved that the split sfGFP system would not be a good candidate for cell-free biosensor design and development. In contrast, split mNG emerged as a promising toolbox for the CFS biosensor, given its contingent conjoining in the CFS and its nonfluorescence in the absence of the 11th segment. Yet, it is noteworthy that even upon optimization of the gene concentration ratio in the CFS, its fluorescence was substantially diminished to only 6% of the full-length mNG. Aiming to enhance this complementation, we employed SynZip peptide linkers—added to the termini of the two protein segments. The addition of the SynZip linkers resulted in a 73.6% fluorescence signal compared with the full-length mNG and a 4.8-fold increase in expression compared with the mNG split system without the SynZip linkers. However, a reduction in overall system activity was observed upon introduction of the purified mNG 1–10 segment, resulting in a 28–66% decline in protein expression, thwarting our goal of increasing total fluorescent output. With optimizations, our goal could possibly still be reached, and the split system was utilized as a higher output reporter protein for CFS sensors. In conclusion, the mNG split system with the SynZip peptide linkers developed in this study has substantial potential to serve as a robust reporter for the cell-free biosensors that are required to synthesize complex gene circuits at the beginning of the reaction, allowing them to sense low levels of analyte before the reaction ceases.

## Methods

### Strains and Plasmids

*Escherichia coli* strains Subcloning Efficiency *DH5α* [Genotype F^–^ Φ80*lac*ZΔM15Δ(*lac*ZYA-*arg*F) U169 *rec*A1 *end*A1 *hsd*R17(r_K_^–^, m_K_^+^) *pho*A *sup*E44 *thi*-1 *gyr*A96 *rel*A1 λ-] and BL21(DE3) star [Genotype F^–^*ompT hsdS*_B_ (r_B_^–^ m_B_^–^) *gal dcm rne131* (DE3)] were used for cloning and a source of the cell extract, respectively (Invitrogen, Waltham, MA). The *E. coli* cells were grown in either Luria–Bertani (LB) media (10 g/L tryptone, 5 g/L yeast extract, and 10 g/L sodium chloride in Milli-Q water) or 2xYTPG media (16 g/L tryptone, 10 g/L yeast extract, 5 g/L sodium chloride, 7 g/L potassium phosphate dibasic, 3 g/L potassium phosphate monobasic, pH 7.2, and 0.1 M glucose in Milli-Q water).

Plasmids were assembled by using the Gibson assembly. Split mNG genes were obtained from the Addgene. The 1–10 segment sequence was obtained from pSFFV_mNG3K(1–10) (Plasmid #157993)^7^ and inserted into pETBlue1 with a N-terminus histidine tag. The 11th segment was taken from pET_mNG2(1–10)_32aalinker_mNG2(11) (Plasmid #82611)^6^ and inserted into pJL1. Split sfGFP was created by splitting the 11th β-strand sequence in pJL1-sfGFP from the 1–10 segments and inserting it into the pJL1 vector. The 1–10 segment sequence was inserted into pETBlue1 with an N-terminal histidine tag. SynZip linker sequences were later added by inserting the respective genes into the pETBlue1 and pJL1 vectors with SynZip linkers already present. Plasmids were transformed into DH5α electrocompetent cells for cloning and plasmid purification (Qiagen Plasmid Midi Kit) for sequencing and a cell-free reaction.

### Whole-Cell Protein Expression and Fluorescent Measurement

The pETBlue1 plasmids containing the 1–10 and the 11th segment genes and full-length sfGFP and mNG in pJL1 were transformed into *E. coli* BL21(DE3) star competent cells via electroporation. A colony (BL21, 1–10 segment harboring plasmid) was selected and grown overnight in 5 mL of LB for the second transformation of the plasmid harboring the 11th segment, while the other colonies were saved for 1–10 segment-only overexpression. The next day, the cells were harvested and washed in a series of 80% glycerol solution (4 °C, 5000 rpm, 5 min). The OD_600_ of the final competent cell was determined to be 0.8–1.0. 50 μL of the competent cell was used to transform the 11th segment harboring plasmid pJL1. The cells were selected under carbenicillin and kanamycin antibiotics, working concentration at both 50 μg/mL. Three green fluorescent protein gene and segment harboring cells (full-length, 1–10 segment only, 1–10 with the 11th segments) were cultured overnight in 5 mL of LB. The overnight cultures were then inoculated to 50 mL of LB in a 1:500 ratio. At OD_600_ 0.5, the cells were induced with 1 mM IPTG. The whole-cell fluorescence was read when the OD600 reached 3.0 with a Synergy HTX multimode microplate reader (BioTek, Winooski, VT, USA). Excitation and emission wavelengths were 485 and 528 nm, respectively.

### Cell Extract Preparation and Cell-Free Protein Synthesis

Cell extract was prepared as described previously.^13,14^ Briefly, an overnight cultured *E. coli* BL21(DE3) star in LB media was inoculated to sterilized 1 L 2xYTPG media in a 2.5-L baffled Tunair shake flask, and the cells were cultured at 37 °C with vigorous shaking at 250 rpm. T7 RNA Polymerase expression was induced at OD 0.5 with 1 mM of isopropyl β-D-1-thiogalactopyranoside (IPTG). Cells were harvested at the midexponential phase of OD_600_ at 3.0. Cells were harvested (4 °C, 5000 rpm, 10 min), and washed and resuspended in Buffers A and B, and lysed using sonication following the previous study.^9^ The lysate was centrifuged at 12,000*g* in 4 °C for 10 min, and the supernatant was collected as cell extract. The cell extract was aliquoted, flash-frozen in liquid nitrogen, and stored in a −80 °C freezer until use.

CFS components, including *E. coli* total tRNA mixture (from strain MRE600) ATP, GTP, CTP, UTP, Phosphoenolpyruvate, 20 amino acids, and other materials, were purchased from Sigma-Aldrich (St. Louis, MO), Alfa Aesar (Haverhill, MA), and Fisher Scientific (Hampton, NH). Cell-free protein synthesis reactions were carried out, as mentioned previously.^9^ The reaction volume was 15 μL with the following components: 1.2 mM ATP; 0.85 mM each of GTP, UTP and CTP; 34.0 μg/mL L-5-formyl-5, 6, 7, 8-tetrahydrofolic acid (folinic acid); 170.0 μg/mL of *E. coli* tRNA mixture; 130 mM potassium glutamate; 10 mM ammonium glutamate; 12 mM magnesium glutamate; 2 mM each of 20 amino acids; 57 mM of HEPES buffer pH 7.2; 0.4 mM of nicotinamide adenine dinucleotide (NAD); 0.27 mM of coenzyme A; 4 mM of sodium oxalate; 1 mM of putrescine; 1.5 mM of spermidine; 33 mM phosphoenolpyruvate (PEP); and 27% v/v of cell extract. Plasmid DNA was added at a concentration of 8 nM, unless otherwise noted. Cell-free protein synthesis reactions were carried out for 20 h to ensure completion at 30 °C.

### Protein Purification

A colony was selected and grown overnight at 37 °C in 5 mL of LB media with shaking at 250 rpm. The next day, the culture was inoculated into a larger LB medium culture of 500 mL at a 1:500 ratio. The cells were then cultured at 37 °C with shaking at 300 rpm in a 2.5 mL Tunair shake flask. Once the culture reached OD_600_ 0.5, a 1 mM final concentration of IPTG was added to the culture to induce T7 RNA polymerase expression and subsequent expression of 1–10 segment. The culture flask was then moved to a 20 °C incubator (250 rpm), and the cells were cultured overnight and harvested in the morning. His-tag purification was performed using the harvested cell pellets and the Qiagen Ni-NTA resin, followed by the manufacturer’s protocol. The resin was washed five times with 40 mM imidazole in wash buffer and eluted with 250 mM imidazole in elution buffer. The eluate was concentrated with 10 kDa MWCO Amicon centrifugal filters. The buffer was replaced with a storage buffer compatible with cell-free reaction. Storage buffer consisted of 20 mM sodium phosphate, pH 7.7, 1 mM EDTA, 1 mM DTT, 5% glycerol, and 100 mM NaCl. The purified protein was then aliquoted to small aliquots of 25 uL and flash-frozen in liquid nitrogen. This storage method retained the activity of the purified protein. Once the sample was thawed, it was not refrozen and saved to use again to retain the activity.

### Protein Analysis

The relative fluorescence units (RFU) of the synthesized fluorescent proteins were measured by the multiwell plate fluorometer (Synergy HTX, BioTek, Winooski, VT). Five μL portion of the cell-free synthesized fluorescent protein and 45 μL of Milli-Q water were mixed in a 96-well half area black plate (Corning Incorporated, Corning, NY). The plate was mixed in the plate reader orbitally at medium speed for 15 s and read at a height of 1.5 mm with a gain of 50. The excitation and emission spectra are 485 and 528 nm, respectively. The cell-free synthesized protein was visualized by Coomassie blue staining after protein gel electrophoresis using precasted 4–12% Bis-Tris gradient gel (Invitrogen, Waltham, MA). Purified protein concentration was measured by the Bradford Assay.

### Statistical Analysis

Statistical analyses were conducted using GraphPad Prism 8.4.3 (GraphPad Software) with a 5% significance level. For the parametric analysis of data from the quantification of the synthesized protein, two-way ANOVA followed by Dunnett’s test was used.

## Supporting Information Available

**Figure S1**: Split segments (1–10, 11th, and full length) expressed in the cell-free system; **Figure S2**: Purified mNeonGreen 1–10 segment (N-His_mNG_1–10_SynZip (31.21 kDa)); **Table S1**: DNA sequences (5′ - 3′) used in this study (PDF)
