# Supplementary material for: Expanding the Cell-Free Reporter Protein Toolbox by Employing a Split mNeonGreen System to Reduce Protein Synthesis Workload
Source: ACS Synth Biol. 2024 Jun 5;13(6):1663–8. doi: 10.1021/acssynbio.3c00752 (PMC11197088; doi:10.1021/acssynbio.3c00752)
Supplement: Supplementary file 1 — sb3c00752_si_001.pdf [file sb3c00752_si_001.pdf]

Supporting Information

Expanding the Cell-Free Reporter Protein Toolbox by Employing a Split mNeonGreen System to Reduce Protein Synthesis Workload

Caroline E. Copeland[a,†], Chloe J. Heitmeier[a,†], Khoa D. Doan[a], Shea C. Lee[a], Kassidy B. Porche[a], and Yong-Chan Kwon[a,b,*]

[a] Department of Biological and Agricultural Engineering, Louisiana State University, Baton Rouge, LA 70803, USA

[b] Louisiana State University Agricultural Center, Baton Rouge, LA 70803, USA

[†] These authors contributed equally.

[*] Corresponding Author: E-mail address: yckwon@lsu.edu (Yong-Chan Kwon)

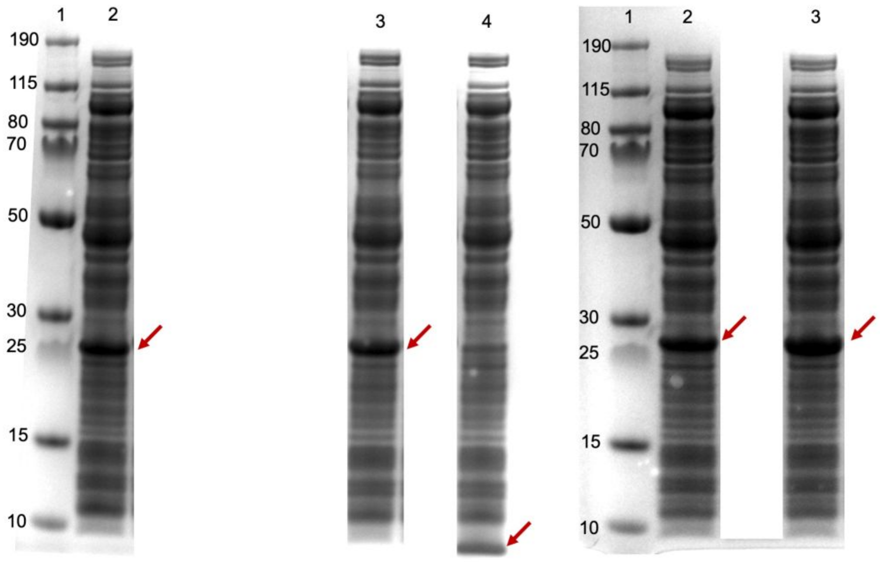

Figure S1. Segments expressed in the cell-free system. (Left gel) Lane 1: protein marker, Lane 2: split mNG 1-10 segment, Lane 3: full-length mNG, Lane 4: split mNG11_SynZip. (Right gel) Lane 1: protein marker, Lane 2: sfGFP 1-10 segment, Lane 3: full-length sfGFP.

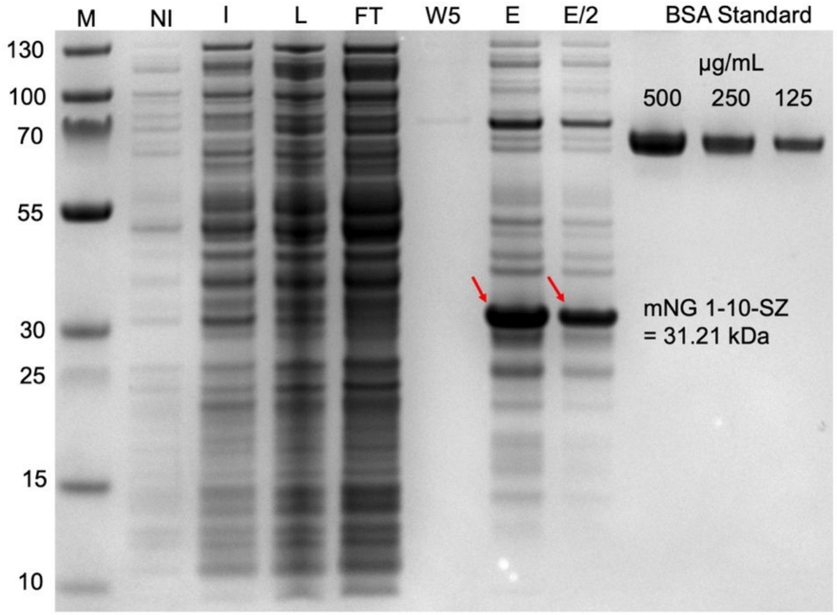

Figure S2. Purified N-His_mNG_1-10_SynZip (31.21 kDa). Lane 1: protein marker, Lane 2: non-induced cell culture sample, Lane 3: induced cell culture sample, Lane 4: lysate of harvested sample, Lane 5: flow-through, Lane 6: last wash (5th), Lane 7: elution, Lane 8: elution diluted by half volume, Lane 9: BSA standard 500 µg/mL, Lane 10: BSA standard 250 µg/mL, Lane 11: BSA standard 125 µg/mL.

Table S1. DNA sequences (5' - 3') used in this study.

SynZip17 + linker	agcatcgcggcgaccctggagaacgatctggcgcgtctggaaaacgaaaacgctcgtttggaaaaagacatcgcgaacctggaacgtgacctggcgaaactggagcgtgaagaagcgtacttcggcggtagtggtggca
SynZip18 + linker	agaacggaggttcaggtggtggatccaacgaaaaagaagaactgaaatccaaaaaagcggaactgcgcaaccgtatcgaacagctgaaacagaaacgtgaacaactgaagcagaaaatcgcgaacctgcgtaaagaaatcgaagcttacaaa
sfGFP	atgagcaaaggtgaagaactgtttaccggcgttgtgccgattctggtggaactggatggcgatgtgaacggtcacaaattcagcgtgcgtggtgaaggtgaaggcgatgccacgattggcaaactgacgctgaaatttatctgcaccaccggcaaactgccggtgccgtggccgacgctggtgaccaccctgacctatggcgttcagtgttttagtcgctatccggatcacatgaaacgtcacgatttctttaaatctgcaatgccggaaggctatgtgcaggaacgtacgattagctttaaagatgatggcaaatataaaacgcgcgccgttgtgaaatttgaaggcgataccctggtgaaccgcattgaactgaaaggcacggattttaaagaagatggcaatatcctgggccataaactggaatacaactttaatagccataatgtttatattacggcggataaacagaaaaatggcatcaaagcgaattttaccgttcgccataacgttgaagatggcagtgtgcagctggcagatcattatcagcagaataccccgattggtgatggtccggtgctgctgccggataatcattatctgagcacgcagaccgttctgtctaaagatccgaacgaaaaacgggaccacatggttctgcacgaatatgtgaatgcggcaggtattacgtggagccatccgcagttcgaaaaataa
sfGFP 1-10 segment	atgcatcatcaccatcaccacattgaagatggccgtagcaaaggtgaagaactgtttaccggcgttgtgccgattctggtggaactggatggcgatgtgaacggtcacaaattcagcgtgcgtggtgaaggtgaaggcgatgccacgattggcaaactgacgctgaaatttatctgcaccaccggcaaactgccggtgccgtggccgacgctggtgaccaccctgacctatggcgttcagtgttttagtcgctatccggatcacatgaaacgtcacgatttctttaaatctgcaatgccggaaggctatgtgcaggaacgtacgattagctttaaagatgatggcaaatataaaacgcgcgccgttgtgaaatttgaaggcgataccctggtgaaccgcattgaactgaaaggcacggattttaaagaagatggcaatatcctgggccataaactggaatacaactttaatagccataatgtttatattacggcggataaacagaaaaatggcatcaaagcgaattttaccgttcgccataacgttgaagatggcagtgtgcagctggcagatcattatcagcagaataccccgattggtgatggtccggtgctgctgccggataatcattatctgagcacgcagaccgttctgtctaaagatccgaacgaaaaataa
sfGFP 11th segment	atgcgggaccacatggttctgcacgaatatgtgaatgcggcaggtattacgtaa
mNeonGreen (E. coli codon optimized)	atggcaagtctacccgctacacacgaattacacatcttcggtagtattaacggggtggattttgatatggttggtcagggtactggaaacccgaatgacggctatgaggaactgaacctgaagtccaccaaaggtgatctgcaattctctccgtggattctggttccgcatatcggttacggcttccatcaatatttaccgtatccagatggcatgagcccatttcaggcggccatggtcgacggctctggttaccaagtgcatagaaccatgcagttcgaggacggcgcgagcctgacggtgaactaccgctacacctacgagggttcccacatcaaaggcgaagcgcaggtgaaaggtactggcttcccggcagacggtccggttatgaccaatagcttgaccgcggctgactggtgccgttcgaagaagacgtacccgaatgacaaaaccattatctccaccttcaagtggagctataccaccggcaacggtaaacgttaccgcagcactgcgcgtaccacatacaccttcgccaaaccgatggcagctaattatttgaagaaccagccgatgtatgtctttcgtaaaacggagcttaagcacagcaagaccgagctcaactttaaagaatggcaaaaggcgtttaccgatgttatgggtatggatgaactgtataaatggtcccatccgcagtttgaaaagtaa
mNeonGreen 1-10 segment	atgcatcatcaccatcaccacattgaagatggccgtgtgagcaagggtgaggaggataacatggcctctctcccagcgactcatgagttacacatctttggctccatcaacgatgtggactttgacatggtgggtcagggtaccggcaatccaaatgaaggttatgaggagttaaacctgaagtccaccaagggcgacctccagttctccccctggattctggtccctcatatcgggtatggcttccatcagtacctgccctaccctgacgggatgtcgcctttccaggccgccatggtagatggctccggataccaagtccatcgcacaatgcagtttgaagatggcgcctcccttactgttaactaccgctacacctacgagggaagccacatcaaaggagaggcccaggtgatagggactggtttccctgctgacggtcctgtgatgaccaacacg

	ctgaccgctgcggactggtgcatgtcgaagatgacttaccccaacgacaaaaccatcatcagtacctttaagtggagttacatcactgtaaatggcaaacgctaccggagcactgcgcggaccacctacacctttgccaagccaatggcggctaactatctgaagaaccagccgatgtacgtgttccgtaagacggagctcaagcactccatgtaa
mNeonGreen 11th segment	atgaccgagctcaacttcaaggagtggcaaaaggcctttaccgatatgatgtaa
mNeonGreen 1-10 segment + SynZip 17	atgcatcatcaccatcaccacattgaagatggccgtgtgagcaagggtgaggaggataacatggcctctctcccagcgactcatgagttacacatctttggctccatcaacgatgtggactttgacatggtgggtcagggtaccggcaatccaaatgaaggttatgaggagttaaacctgaagtccaccaagggcgacctccagttctcccccctggattctggtccctcatatcgggtatggcttccatcagtacctgccctaccctgacgggatgtcgcctttccaggccgccatggtagatggctccggataccaagtccatcgcacaatgcagtttgaagatggcgcctcccttactgttaactaccgctacacctacgagggaagccacatcaaaggagaggcccaggtgatagggactggtttccctgctgacggtcctgtgatgaccaacacgctgaccgctgcggactggtgcatgtcgaagatgacttaccccaacgacaaaaccatcatcagtacctttaagtggagttacatcactgtaaatggcaaacgctaccggagcactgcgcggaccacctacacctttgccaagccaatggcggctaactatctgaagaaccagccgatgtacgtgttccgtaagacggagctcaagcactccatggagaacggaggttcaggtggtggatccaacgaaaaagaagaactgaaatccaaaaaagcggaactgcgcaaccgtatcgaacagctgaaacagaaacgtgaacaactgaagcagaaaatcgcgaacctgcgtaaagaaatcgaagcttacaaataa
mNeonGreen 11th segment + SynZip 18	atgagcatcgcggcgaccctggagaacgatctggcgcgtctggaaaacgaaaacgctcgtttggaaaaagacatcgcgaacctggaacgtgacctggcgaaactggagcgtgaagaagcgtacttcggcggtagtggtggcaagaccgagctcaacttcaaggagtggcaaaaggcctttaccgatatgatgtaa
